# Evaluation of a skin self examination attitude scale using an item response theory model approach

**DOI:** 10.1186/s12955-014-0189-x

**Published:** 2014-12-24

**Authors:** Ngadiman Djaja, Pip Youl, Joanne Aitken, Monika Janda

**Affiliations:** School of Public Health, Queensland University of Technology, Brisbane, QLD Australia; National Health and Medical Research Council Centre for Research Excellence in Sun and Health, Institute of Health and Biomedical Innovation, Queensland University of Technology, Brisbane, Australia; Cancer Council Queensland, Brisbane, QLD Australia; Griffith Health Institute, Griffith University, Brisbane, Australia

**Keywords:** Skin cancer, Skin self-examination, Attitude scale, Item response theory, Rating scale, Rasch model

## Abstract

**Introduction:**

The Skin Self-Examination Attitude Scale (SSEAS) is a brief measure that allows for the assessment of attitudes in relation to skin self-examination. This study evaluated the psychometric properties of the SSEAS using Item Response Theory (IRT) methods in a large sample of men ≥ 50 years in Queensland, Australia.

**Methods:**

A sample of 831 men (420 intervention and 411 control) completed a telephone assessment at the 13-month follow-up of a randomized-controlled trial of a video-based intervention to improve skin self-examination (SSE) behaviour. Descriptive statistics (mean, standard deviation, item–total correlations, and Cronbach’s alpha) were compiled and difficulty parameters were computed with Winsteps using the polytomous Rasch Rating Scale Model (RRSM). An item person (Wright) map of the SSEAS was examined for content coverage and item targeting.

**Results:**

The SSEAS have good psychometric properties including good internal consistency (Cronbach’s alpha = 0.80), fit with the model and no evidence for differential item functioning (DIF) due to experimental trial grouping was detected.

**Conclusions:**

The present study confirms the SSEA scale as a brief, useful and reliable tool for assessing attitudes towards skin self-examination in a population of men 50 years or older in Queensland, Australia. The 8-item scale shows unidimensionality, allowing levels of SSE attitude, and the item difficulties, to be ranked on a single continuous scale. In terms of clinical practice, it is very important to assess skin cancer self-examination attitude to identify people who may need a more extensive intervention to allow early detection of skin cancer.

## Introduction

Melanoma is the fourth most common cancer among men and women in Australia. Men aged 50 years or older are more likely than other groups to be diagnosed with thick melanomas and have the highest mortality [1]. Skin self-examination (SSE) has been shown to increase the detection of thin melanoma [2-4]. A case-control study in the United States found a 60% reduced risk of melanoma mortality (OR 0.37; 95% CI = 0.16-0.84) in people who examined their own skin [4]. While the US Preventive Services Task Force currently does not recommend population-based screening for skin cancer due to the absence of randomised trials investigating the mortality benefit of screening [5], the American Cancer Society does recommend that adults perform SSE monthly [6] and Australian Cancer Councils suggest SSE at three-monthly intervals [7].

SSE may be one method of identifying suspicious skin lesions early, particularly given that patients are more likely to detect their own melanomas [8]. A large case-control study conducted in Queensland, Australia found that melanomas detected during deliberate SSE compared to those found incidentally, were thinner [9]. As about half of all melanomas occur on parts of the body that are difficult to see (especially the back) [10], it has been suggested that whole-body SSE is necessary to optimise melanoma detection rate [11].

While melanoma incidence and mortality is highest in men 50 years or older, this group is less likely to detect their own melanomas and were less likely to undergo whole-body clinical skin examination compared to other population groups [12,13]. Both of which could contribute to their higher melanoma mortality rates. The increased risk of thick melanoma in this group may be due, at least in part, to low awareness and uptake of early detection behaviours, including SSE.

Several aspects of SSE are under-researched, and few studies have measured factors which may contribute to whether or not people conduct SSE. One study by Manne and Lessin [14], who developed a 17-item SSE benefits and barriers scale, found only barriers (but no benefits) were associated with SSE performance in melanoma survivors. The authors suggested that melanoma survivors rely strongly on their doctors’ recommendation, minimising the impact of their personal attitudes, and that further assessment among the general population is needed. Swetter et al. [15] found that SSE awareness (defined as having heard about the ABCD rule, reading about skin cancer detection, and requesting information about skin cancer detection from doctor) of female spouses of men with melanoma was significantly higher than that of the men themselves.

We previously used several attitude or outcome expectation items within a large study of melanoma screening, and found that positive attitudes was strongly associated with intention to conduct SSE in the future [16]. However the psychometric qualities of the measure as a whole have not been assessed.

Measurement of subjective and latent constructs like SSE attitudes requires rigorously developed and tested instruments in order to obtain data of the highest possible quality. While in the past questionnaire quality including reliability and validity was often assessed using classical psychometric approaches, increasingly the advantages of item response theory (IRT) methods, including allowing more precise estimates, assessment of unidimensionality, adaptive testing and assessment of differential item functioning have been recognised. IRT methods are now applied to measurement tools across a wide variety of health outcomes [17-21]. It was the aim of this study to evaluate the measurement properties and unidimensionality of the SSE attitude scale using a Rasch modelling approach.

## Methods

To examine measurement properties of the SSE attitudes scale we used data collected from the Skin Awareness study [22]. The primary aim of that study was to examine the impact of a video-delivered intervention with two mailed reminder postcards compared to a written-materials- only control group on the prevalence of SSE in men aged 50 years or older. The primary hypothesis was that the prevalence of SSE in the video intervention group would increase by at least 10% more than in the control. A 10% increase was determined as the minimal change deemed to be clinically significant. Approval for this study was obtained from the Queensland University of Technology ethics committee, and the trial was registered with the Australian New Zealand Clinical Trials Registry (ANZCTR N12608000384358). Trial methods and baseline participant characteristics as well as primary and secondary outcomes have previously been reported in detail [22-24].

### Study population

In total, 5000 potential participants (men aged 50 or older) were randomly selected from the Australian electoral roll (enrolling to vote is compulsory in Australia), of which 2899 potential participants with a valid telephone number were contacted by mail. The study pack included a letter of invitation and a colored brochure featuring a well-known sports and TV personality, with follow-up of non-respondents via one postal reminder and up to two follow-up phone calls. Men who were too ill, could not speak English, or had a previous history of melanoma were excluded. The overall consent rate was 37% (969 of 2610 eligible); however 39 men withdrew before the study began, leaving a final sample of 930 men who were randomised to the control or intervention condition. Men completed telephone interviews at baseline, at 7 and 13 months after receiving either the video intervention or written brochures only control package.

For the present analysis, we used data from 831 men who completed the 13-month assessment time point. Similar to factor analysis, where a minimal sample size of 10 is required per item by convention, a minimum sample size of 250 is generally requested for analyses such as those conducted here [25].

### Skin self-examination attitude scale

The skin self-examination attitude scale (SSEAS) developed, and previously used, in a large community-based pilot trial of skin cancer screening [16], and was modified for the Skin Awareness study to include items measuring SSE outcome expectancy and planning for future SSE. The SSEAS includes a list of 10 items, answered on a five point Likert scale ranging from strongly disagree, disagree, unsure, agree, and strongly agree (all items listed in Table 1). The total score of the SSEAS can vary between 0 and 40, where 0 indicates low and 40 high SSE attitudes. Good reliability for the scales was found when assessing its internal consistency (Cronbach alpha .80).

**Table 1:** **Item total correlation, fit statistics and item difficulty for the 10-item skin self-examination attitude scale**

		**Item total correlation**	**Mean square**		**Item difficulty (SE)**
			**Infit**	**Outfit**	
SSE_1	It is important to check my skin for skin cancer even if I have no symptoms	0.457	0.92	1.03	- 0.58 (0.07)
SSE_2*	I think checking my skin would make me anxious*	0.081	-	-	-
SSE_3	Checking my skin regularly is a priority for me	0.526	1.05	1.25	0.54 (0.05)
SSE_4	I think I could find something suspicious on my skin if it was there	0.495	0.99	1.06	0.23 (0.06)
SSE_5	If I saw something suspicious on my skin, I’d go to the doctor straight away	0.446	1.03	1.06	-0.36 (0.06)
SSE_6	I am confident in a doctor’s ability to diagnose skin cancer	0.373	1.20	1.32	-0.07 (0.06)
SSE_7**	I have made plans on when to examine my own skin*	0.461	-	-	-
SSE_8	I am confident that I can take up examining my own skin again even if I have not looked at my skin in the past few months	0.579	0.82	0.81	0.18 (0.06)
SSE_9	I am able to keep examining my own skin regularly, even if I have no one to help me	0.474	1.03	1.34	0.53 (0.06)
SSE_10	If I regularly examine my skin, then I am helping to look after my own health	0.582	0.75	0.67	-0.46 (0.07)

*Item was removed due to low item total correlation.

**Item was removed during calibration due to fit statistics beyond acceptable range.

### Data analysis

To test the measurement quality of the SSEAS beyond classical test theory, item response theory (IRT) modelling was applied. In brief, IRT model measures the relationship between an individual’s ability and an item difficulty, and models this as a probabilistic function. Specifically, raw data from a rating scale are converted to an “equal interval scale” in logits (log odd units), reflecting the item difficulty and individual’s ability [26,27]. Data were analysed using the Winsteps Rasch Measurement [28]. To analyse the SSEAS, with 5 answer options per item, the polytomous Rasch Rating Scale Model (RRSM) was used.

The following data quality parameters were assessed:

### Dimensionality analysis

We assessed whether the data derived from the men’s answers fitted the Rasch model in order to assess unidimensionality of the underlying trait. To assess the fit of the data to the Rasch model, item difficulty and fit statistics were calculated for each item.

### Item difficulty

The difficulty of each SSEA item is its point on SSEA logits – when SSEA is expressed as a unidimensional continuum. For polytomous scales including the SSEAS, this is the point at which each answer category has a 50% probability of being endorsed. Winsteps ranks the items in a hierarchical order based on their item difficulty. The item at the top has high item difficulty and thus is difficult for people to endorse; the item at the bottom of the rank is an easy-to-endorse item. Item difficulty is calculated in logits and placed on a linear interval continuum. The higher the logit is, the more the item measures at a high SSEA difficulty level.

### Item fit statistics

To determine item fit statistics, infit and outfit mean square (MNSQ) statistics were calculated, which specify how well each item fits the Rasch model. Infit and outfit MNSQ values should range from 0.6 to 1.4 [29]. These fit statistics represent the difference between expected responses and observed responses. An item perfectly fits with the model if they have MNSQ of 1. Values less than 1.0 (overfit) show the model predicts the data too well - causing summary statistics (e.g: reliability), to report inflated statistics. Meanwhile values greater than 1.0 (underfit) show unmodeled noise (there is other source of variance in the data) - these will degrade measurement.

The infit and outfit MNSQ represents the unstandarised degree of fit of data observation to the Rasch model expected responses. While the infit MNSQ is sensitive to unexpected patterns, the outfit MNSQ statistic is more sensitive to outliers.

### Differential item functioning (DIF)

DIF was assessed to examine if the intervention condition had an effect on the hierarchy of item difficulties. Rasch assumes the hierarchy of the items to be the same across groups: it should work uniformly, irrespective of groups, in our case, for men in the intervention and control groups. For example, if an item is invariant across groups, the item with the lowest difficulty on the SSEA continuum for the intervention group has also the lowest difficulty for the control group. Instead of calculating the item difficulties for the whole group, in DIF analysis they are now calculated separately (per group). The current study used a multi-step method of initially flagging items for potential DIF using the Mantel chi-square statistic, followed by confirmation of DIF with two other tests (Standardized Liu-Agresti Cummulative Common Log-Odds Ratio (LOR Z) and Standardized Cox’s Noncentrality Parameter (COX Z)). All MH-based statistics were computed using DIFAS 5.0 [30].

## Results

SSEAS data was available from 831 participants, 411 (49.5%) control group participants with a mean SSEAS score of 4.1 (SD 0.49) and 420 (50.5%) intervention group participants (mean SSEAS score of 4.1 (SD 0.50).

### Unidimensionality

The Rasch analysis showed good reliability. Item reliability (replicability of item placements along the scale) was 0.98 and person reliability was 0.68. Individual item difficulty level ranged from – .58 to .54 logits, with a mean ± standard deviation (SD) of 0 ± 0.41. Whereas person measures had a mean ± SD of 1.71 ± 1.40, indicating that the items did not adequately target the SSEA levels of this sample. Results of the unidimensionality analysis are shown in Table 1.

### Item difficulty

Item difficulty estimates found that the easiest item to endorse for the participants was item SSE_1 (- 0.58): “It is important to check my skin for skin cancer even if I have no symptoms” while the most difficult item to endorse was item SSE_3 (0.54): “Checking my skin regularly is a priority for me”.

Items SSE_3 and SSE_9 both had about the same item difficulty of 0.54 logits and 0.53 logits, with evidence for overlap between the items and thus redundancy of items. In addition, items SSE_4 (0.23) and SSE_8 (0.18) measure a similar level of SSEA evidenced by a separation distance of only 0.05 logits.

**Figure 1:**
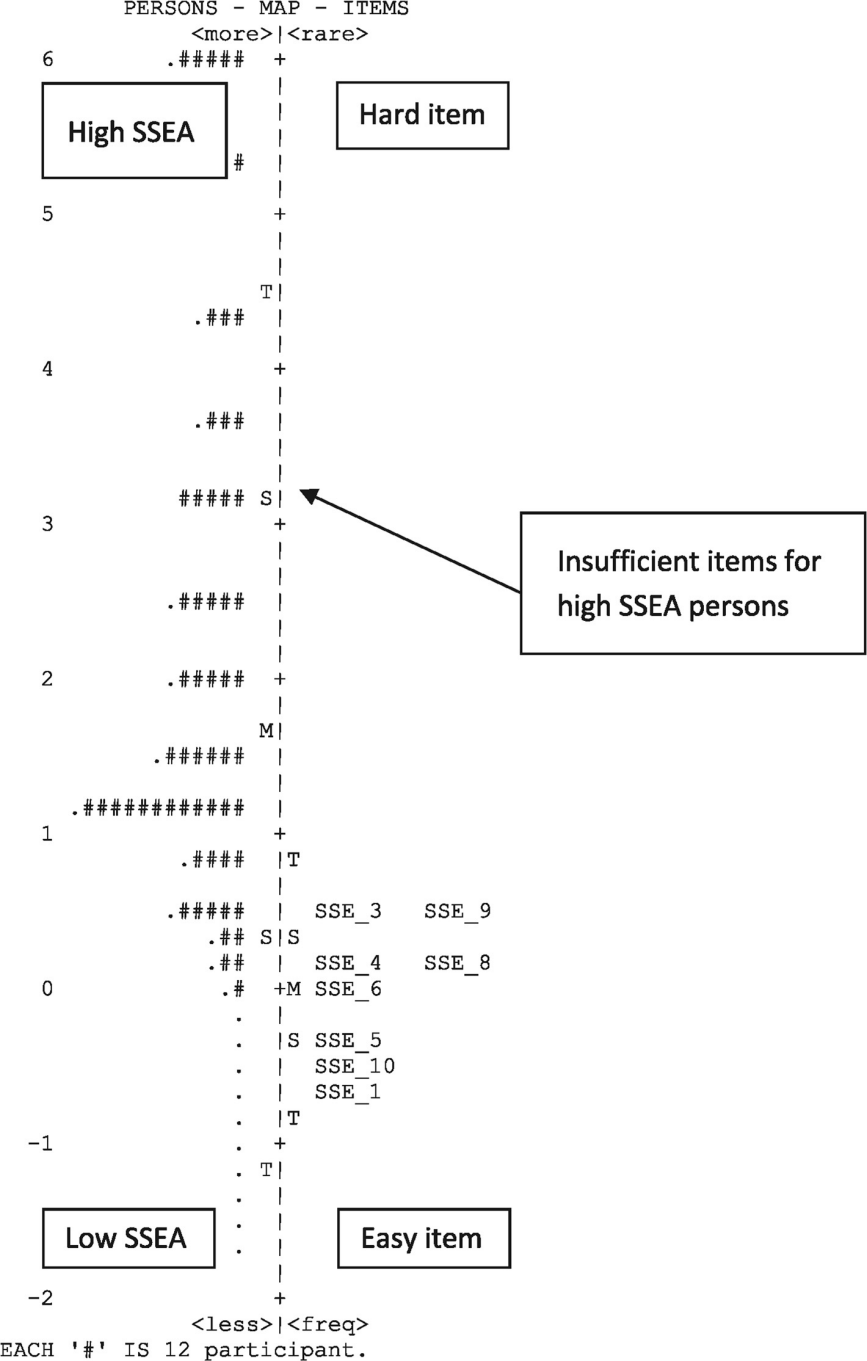
**Wright map/item person map of Skin Self-Examination Attitude Scale with the mean theta of persons on the left and mean theta of items on the right.**

We also assessed the spread of item difficulty using the item-person map (Wright map) displayed in Figure 1. This map indicates both the distribution of participants’ SSEA propensity scores, and item difficulty levels. Both the items and responses are displayed on a logit scale; respondents with the same SSEA propensity scores as the item difficulty have a 50% chance of endorsing the item. The left hand side of Figure 1 shows the distribution of respondents’ level of SSEA, people with a higher SSEA are placed in the higher position and people with lower SSEA are placed in the lower positions. The right hand side shows the distribution of item calibrations, items reflecting higher SSE attitude are placed in higher position and items reflecting a lower SSEA level are placed in lower positions.

M is the mean value (the default value of participants mean is set to 0), while S labels one standard deviation and T labels two standard deviations of the item and person distribution. The map shows that the participants’ average SSEA mean was 1.71 logit above the items’ mean, implying that participants have a high level of SSEA.

### Content coverage and item targeting

A ceiling effect was evident in the results displayed in Figure 1, with many participants located in the upper part of the map, and few items located in the corresponding level. The SSEA of this sample was higher than that reflected in the items. The mean of item measures was more than 1 standard deviation lower than the mean of person measures, which indicates that all items were easily endorsed by this sample, and additional items with greater difficulty are needed to complement the scale.

### Item fit statistics

After an iterative process of calibration, all items of the SSEAS except SSE_2: “I think checking my skin would make me anxious” and SSE_7: “I have made plans on when to examine my own skin” were found to have inadequate MNSQ infit and outfit outside the recommended values 0.6 and 1.4 [26] (Table 1) and overall did not met the fit criteria. SSE_2 also did not contribute to the measurement of a unidimensional construct.

### Differential item functioning (DIF) assessment

The eight item SSEA scale was used to assess DIF by group condition (DVD intervention and control). Result of DIF analysis is presented in Table 2, and revealed that none of the eight items showed DIF according to participants’ group condition.

**Table 2:** **DIF statistics for the 8-item skin self-examination attitude scale**

	**Mantel** ^**1**^	**LORZ** ^**2**^	**COX Z** ^**2**^
SSE_1	0.870	0.954	0.931
SSE_3	0.268	0.524	0.519
SSE_4	0.719	-0.842	-0.849
SSE_5	0.177	-0.422	-0.419
SSE_6	0.238	-0.485	-0.491
SSE_8	0.063	0.253	0.250
SSE_9	0.814	-0.904	-0.903
SSE_10	1.540	1.238	1.240

^1^Critical values of this statistic are 3.84 for a Type I error rate of 0.05.

^2^A value greater than 2.0 or less than –2.0 may be considered evidence of the presence of DIF.

## Discussion

Regular monthly or 3-monthly SSEis currently recommended by a number of cancer control agencies, particularly for those at high risk such as older men who carry the greatest skin cancer burden of skin cancer. SSE could improve skin awareness and rapid clinical skin examination. In combination this has potential to reduce the physical burden, including mortality, caused by late diagnosis of melanoma [31,32]. Studies have shown that melanomas detected during a deliberate SSE rather than found accidentally are thinner [2,33]. Attitudes towards SSE form an important component in explaining the likelihood of conducting an SSE [34].

IRT has been used widely in evaluation education and health measures [19,35,36]. The current study used IRT analysis to further assess the psychometric properties of the SSEAS. Data were analysed using the Rasch Rating Scale Model [37], which has ideal metric properties for ranking an individual’s ability (the level of the attribute measured) along with the item difficulty on a common scale. This Rasch model allows for the comparison of individuals regardless of items used in the measurement [38]. It also enables the generation of a joint measurement (common scale) of items and people, provided that the data is fitted to the model’s requirements.

In this study, the overall fit statistics and reliabilities of the SSEAS were satisfactory. However, the spread of item difficulty was not satisfactory, with most items located on the lower end of the scale. This means the SSEAS will give more accurate information for individuals who have low skin-self-examination attitude. Two items (item SSE_2 and item SSE_7) did not perform as expected and were removed to achieve better fit to the Rasch model expectations. This suggested that those two items may be measuring a different domain of SSE. Item SSE_2: “I think checking my skin would make me anxious” was suspected to measure anxiety rather than attitude. Item SSE_7: “I have made plans on when to examine my own skin” probably measures the planning aspect of SSE. This item could form a separate scale with additional planning items that address the specific aspects of optimal SSE performance (such as having a partner to help, or having available a full size and hand held mirror, or good lighting) have been added.

The distribution of the SSEAS items reflects a wide range of individual differences, with the average level of this trait in the current sample being higher than the average difficulty level of the items. The difficulty level of the 8 items reflected a narrow range of levels of skin self-examination attitude among men ≥ 50 years, thus not allowing for the optimal discrimination of more positive attitude in this sample.

The DIF analysis according to study group showed that the functioning of the 8 items on the SSEAS was consistent, and was considered equally difficult, for both intervention and control groups. The items were sufficiently robust to allow for the assessment of SSE attitudes regardless of the participant’s group. Thus, the answers only quantified the individual’s level of SSE attitude, which was measured according to the difficulty of the items and not because of other constructs explained by the participant’s subgroup.

The present study has some limitations. People with a high risk of skin cancer may feel social pressure to report higher SSEA than others, and this may have resulted in a positive reporting bias to our SSEA score. Although there is no objective measure of SSE, adding a social desirability scale such as the Marlowe-Crowne social desirability scale [39] in future studies could allow assessment of the SSEA scale against this criterion. The addition of more high SSEA items to extend the difficulty range of the measure may also help to improve the scale. Finally, our sample consisted entirely of men aged 50 years or older. Future research should examine whether these results also hold for the broader population including sample from other states in Australia, women and younger age groups.

## Conclusion

Overall, the present study confirms the SSEA scale as a brief, useful and reliable tool for assessing attitudes towards skin self-examination in a population of men 50 years or older in Queensland, Australia. The 8-item scale shows unidimensionality, allowing levels of SSE attitude, and the item difficulties, to be ranked on a single continuous scale. In terms of clinical utility, the skin awareness scale can identify people who may need a more extensive intervention. Clinician can encourage these people to start skin self examination regularly looking for any abnormal growth or unusual changes, so they can have a better chance for a cure.
